# Measurement of the Nucleus Area and Nucleus/Cytoplasm and Mitochondria/Nucleus Ratios in Human Colon Tissues by Dual-Colour Two-Photon Microscopy Imaging

**DOI:** 10.1038/srep18521

**Published:** 2015-12-17

**Authors:** Chang Su Lim, Eun Sun Kim, Ji Yeon Kim, Seung Taek Hong, Hoon Jai Chun, Dong Eun Kang, Bong Rae Cho

**Affiliations:** 1 Department of Chemistry, Korea University, 145 Anam-ro, Sungbuk-gu, Seoul 136-713, Korea; 2 Department of Internal Medicine, Korea University College of Medicine, 73 Inchon-ro, Seoul, 136-705, Korea; 3 KU-KIST Graduate School of Converging Science and Technology, Korea University, 145 Anam-ro, Sungbuk-gu, Seoul 136-713, Korea

## Abstract

We developed two-photon (TP) probes for DNA (ABI-Nu), cytoplasm (Pyr-CT), and mitochondria (BF-MT). We found that ABI-Nu binds to AT in the minor groove, while ABI-Nu and BF-MT are effective for tracking in the cytoplasm and mitochondria, respectively. These probes showed very large effective two-photon action cross section values of 2230, 1555, and 790 Göppert-Mayer units (1 GM  =  10^−50^ cm^4^ s photon^−1^molecule^−1^) at 740 nm with emission maxima at 473, 561, and 560 nm, respectively, in each organelle. Using these probes, we quantitatively estimated the mean nuclear area and the ratios of nuclei to cytoplasm and mitochondria to nuclei in human colon tissues by dual-colour two-photon microscopy imaging within 2  h after biopsy. The mean nuclear area and the nuclei to cytoplasm and mitochondria to cytoplasm ratios increased in the following order: normal colon mucosa <colon adenoma <colon adenocarcinoma. Furthermore, the nuclear areas of these tissues showed significant differences that were well outside of the ranges of experimental errors, indicating the diagnostic potential of this method.

Colon cancer is one of the most common cancers and mainly develops from the colon adenoma-adenocarcinoma sequence^1^. Polyps or tumors found during endoscopy are removed, processed with formalin and paraffin to generate thinly sliced sections, stained with hematoxylin and eosin, and imaged for cancer diagnosis^2^. Pathologists examine the tissue structure and cellular morphometry including the nucleus size and nucleus to cytoplasm (N/C) ratio to ascertain the diagnosis; the entire process requires several days to complete. However, the results are unreliable because nucleus size and N/C ratio are subjective findings and not objectively quantifiable values. Moreover, they are based on a single sectional image, which does not accurately represent all histopathologic features of the *ex vivo* slice. In fact, there are reports regarding the discrepancies and difficulties in the diagnosis of early adenocarcinoma and high grade dysplasia/adenoma because of the different standards used in various locations and inter-observer variations^3^,^4^.

Two-photon microscopy (TPM) is an attractive tool for obtaining more objective and quantitative information for the cellular morphometry of tumour tissues. TPM, which utilizes two near-infrared photons for excitation, is an invaluable tool in biomedical research because it can be used for imaging deep inside live tissues to provide intrinsically high spatial resolution^5^,^6^. Using TPM, hundreds of sectional images of *ex vivo* slices can be obtained, from which the average size of target organelles can be measured. Recently, it was reported that the mitochondria of cancer cells are enlarged and increased in number^7^,^8^, which may be used as a diagnostic measure for cancer. The development of two-photon (TP) probes for the nuclei, cytoplasm, and mitochondria that emit TP-excited fluorescence (TPEF) at widely separated wavelengths may enable the simultaneous estimation of the mean nuclear area (N-area) and the ratios of N/C and nuclei to mitochondria (M/N) using dual-colour TPM imaging, which may serve as a new method for pathologic diagnosis of colon cancer. Recently, a number of TP probes for nuclei^9^,^10^,^11^ and mitochondria^13^,^14^,^15^ have been developed. However, TP probes that meet the requirements described above have not been reported.

Therefore, we developed new TP probes for DNA (ABI-Nu), cytoplasm (Pyr-CT-AM), and mitochondria (BF-MT) (Fig. 1). ABI-Nu was derived from Hoechst 33538, a well-known DNA probe^16^, by introducing a 2-amino-6-naththyl group at the 2-position of the benzimidazole moiety in place of the 4-alkoxyphenyl group with the expectation that the extended conjugation length and increased donor strength would enhance TPEF intensity. We also developed Pyr-CT-AM derived from 1,4-bis(styry)pyrazine as a TP probe for the cytoplasm^17^, and expected that this probe would emit strong TPEF at a much longer wavelength than ABI-Nu and that the acetoxymethyl (AM)-ester derivative would improve cell loading. We then developed BF-MT as a TP probe for mitochondria. We expected that the positively charged and strongly electron-accepting pyridinium ion would localize this probe to the mitochondria and emit TPEF at a wavelength longer than that of ABI-Nu.

Here, we report that ABI-Nu, Pyr-CT, and BF-MT can be used to visualize the nuclei, cytoplasm, and mitochondria in normal colon tissue, adenoma with low grade dysplasia, and adenocarcinoma by dual-colour TPM imaging. Particularly, we found that the N-area and the N/C and M/N ratios were higher in cancerous than in normal tissue, indicating the diagnostic potential of this approach.

## Results

### Synthesis, spectral properties, and water solubilities

The preparation of ABI-Nu, Pyr-CT, and BF-MT is described in the Supporting Information. ABI-Nu, Pyr-CT, and BF-MT showed absorption maxima (λ_max_) at 362 nm (ε = 14,200), 437 nm (ε = 24,180), and 420 nm (ε = 10,200), respectively, in phosphate buffer (10 mM sodium phosphate buffer, pH 7.4, 100 mM NaCl) (Fig. S1). The fluorescence maximum (λ_fl_) of ABI-Nu appeared at 472 nm (Φ = 0.0043), but the emission from Pyr-CT and BF-MT were too weak to permit measurement of their λ_fl_ values in the phosphate buffer. In EtOH, all probes showed appreciable Φ values, with ABI-Nu, Pyr-CT, and BF-MT showing λ_fl_ at 452 nm (Φ = 0.45), 577 nm (Φ = 0.020), and 550 nm (Φ = 0.040), respectively (Table S1). The solubilities of ABI-Nu, Pyr-CT-AM, and BF-MT in phosphate buffer, as determined using a fluorescence method^18^, were 3.0, 4.0, and 6.0 μM (Fig. S2), respectively, which were sufficient for cell staining.

### Binding mode of ABI-Nu with DNA

**Figure 2:**
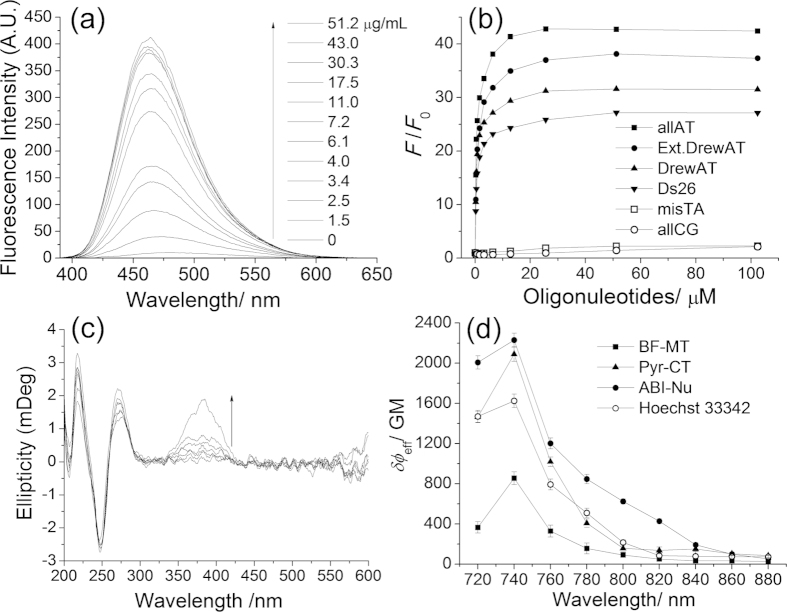
(**a**) One-photon emission spectra of ABI-Nu (1 μM) in PBS in the presence of CT-DNA (0−51.2 μg/mL). (**b**) Fluorometric titration curves obtained by plotting the fluorescence enhancement (*F*/*F*_0_) vs the molar concentration of oligonucleotides. (**c**) CD spectra of allAT (10 μM) in the presence of ABI-Nu (0−64 μM) in PBS. (**d**) Effective two-photon action across sections of Pyr-CT (-▲-), ABI-Nu (●), Hoechst 33342 (-○-), and BF-MT (-◼-) in HeLa cells.

The binding mode of ABI-Nu with DNA was investigated using ultraviolet-visible (UV-Vis), fluorescence, and circular dichroism (CD) spectra. When calf thymus DNA (CT-DNA) was added to ABI-Nu in phosphate buffer, the absorption spectrum was red-shifted (Δλ_max_ = 12 nm), while the emission spectrum was blue-shifted (Δλ_fl_ = 9 nm), with a 30-fold increase in fluorescence intensity (Fig. 2a). Similar results were observed in the presence of double-stranded oligonucleotides containing AT sequences (Table S2), with greater fluorescence enhancement (*F/F*_*o*_) for oligonucleotides with more successive AT sequences (Fig. 2b). AllAT pairs (i.e., 12 AT base pairs) showed the largest enhancement (*F/F*_*o*_ = 43), followed by extended DrewAT (6 successive AT base pairs in the central core with additional AT sequences in the side chain, *F/F*_*o*_ = 38), DrewAT (6 successive AT base pairs in the central core, *F/F*_*o*_ = 32), and ds26 (4 successive AT base pairs in the central core, *F/F*_*o*_ = 27). In contrast, only a slight change in the *F/F*_*o*_ value was observed upon addition of RNA, misTA (a single-stranded oligonucleotide) or allCG (a double-stranded oligonucleotide without an AT sequence (Fig. 2b and S3).

The CD spectra of the oligonucleotide showed duplex DNA signals at 200−300 nm in the presence of ABI-Nu, indicating that the duplex structures of self-complementary oligonucleotides were successfully synthesized (Fig. 2c and S4)^19^. The CD spectra of all oligonucleotides, except allCG, showed strong positive CD signals at 380 nm in the presence of ABI-Nu. Furthermore, the signals were stronger for DNAs with a larger number of successive AT sequences (Fig. 2c and S4).

### TP action cross section and effective TP action cross section

The TP action cross-sections (Фδ) of ABI-Nu, Pyr-CT, and BF-MT were determined by measuring fluorescence and using Rhodamine 6G in MeOH as a reference^20^,^21^. The measurements were conducted in phosphate buffer containing allAT (ABI-Nu), 1,4-dioxane/H_2_O (32/1) (Pyr-CT), and EtOH (BF-MT). We chose these solvents as models for intracellular environments because ABI-Nu can be localized in the DNA and because the TPEF maxima of ABI-Nu (473 nm), Pyr-CT (561), and BF-MT (560 nm) in HeLa cells were similar to their emission maxima in these solvents (472, 560, and 550 nm, respectively) (Table S1). The maximum TP action cross-sections (Фδ_max_) of ABI-Nu, Pyr-CT, and BF-MT were 12 GM at 740 nm, 194 GM at 750 nm, and 13 GM at 750 nm, respectively (Fig. S5 and Table S1).

To quantitatively measure TP brightness, we determined the effective Фδ values (Фδ_eff_) by comparing the TPEF intensities in each organelle in the probe-labelled cells with that of Rhodamine 6G in MeOH (see the Methods section for details). The Фδ_eff_ values of ABI-Nu, Pyr-CT-AM, and BF-MT were 2230, 1555, and 790 GM at 740 nm, indicating that their local concentrations in each organelle were 186, 8, and 60 μM, respectively (Fig. 2d).

To compare the TP brightness of ABI-Nu and Hoechst 33258, we also measured the Фδ_max_ and Фδ_eff_ values of the commercial probe. The values were 10 and 1625 GM at 740 nm, respectively, indicating that the Фδ_max_ and Фδ_eff_ values were larger for ABI-Nu than for Hoechst 33258 and that the local concentration of Hoechst 33258 in the nucleus was approximately 160 μM.

### Photostability, pH dependency, and cytotoxicity

Photostability was determined by monitoring the TPEF intensities at three different positions chosen without bias in the probe-labelled cells. ABI-Nu and Pyr-CT-AM showed high photostability as indicated by the negligible change in TPEF intensity over 1 h, whereas BF-MT showed modest photostability as indicated by the appreciable decrease in TPEF intensity (Fig. S6d–f). However, because only one pulse is irradiated at a given spot during imaging, all probes showed sufficient photostabilities for obtaining TPM images.

pH dependency was evaluated by measuring the fluorescence intensities of ABI-Nu, Pyr-CT, and BF-MT in PBS at pH 5.0−8.0. The fluorescence intensity changed only slightly over this pH range, indicating pH-independency over the biologically relevant pH range (Fig. S7).

Cell viability was measured using a Cell Counting Kit-8 (Dojindo, Kumamoto, Japan) according to the manufacturer’s protocol. All probes showed minimum cytotoxicity under our incubation conditions (Fig. S8).

### TPM imaging

**Figure 3:**
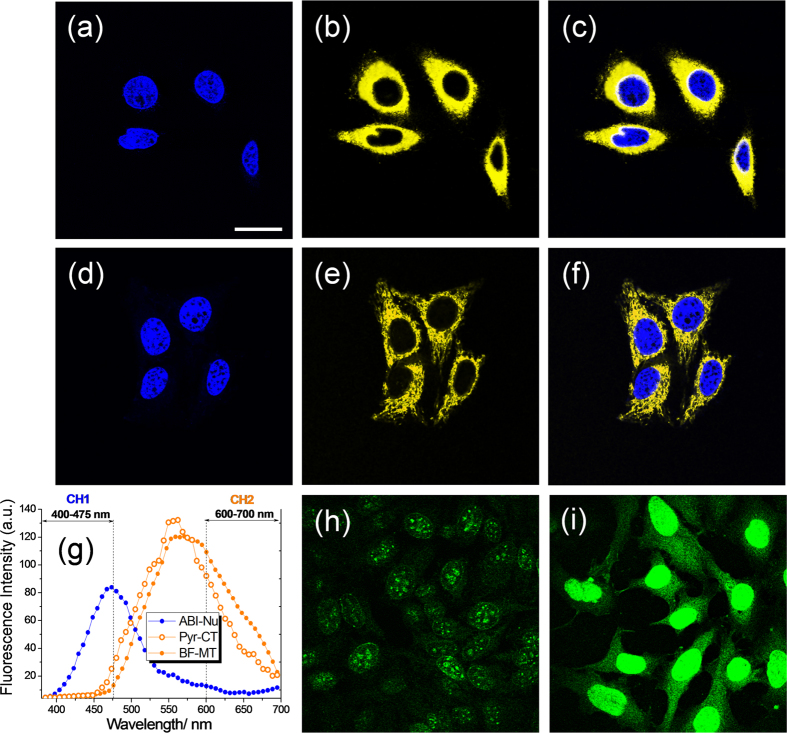
TPM images of HeLa cells co-labelled with (a–c) ABI-Nu (0.5 μM) and Pyr-CT (1.0 μM), and (d–f) ABI-Nu (0.5 μM) and BF-MT (1.0 μM). (**a,b**) TPM images collected at Ch1 (nuclei) and Ch2 (cytoplasm and mitochondria), respectively. (**c,f**) Merged images. (**g**) Two-photon excited fluorescence (TPEF) spectra of HeLa cells labelled with ABI-Nu (blue), Pyr-CT (orange outline), and BF-MT (filled orange), respectively. (**h,i**) TPM images of HeLa cells labelled with ABI-Nu collected at Ch1 after digestion with 0.5 μg/mL each of (**h**) DNase and (**i**) RNase. HeLa cells were incubated with the probes for 30 min at 37 °C. The TPM images were collected with magnification at 100× upon excitation at 750 nm. Scale bar = 30 μm.

To determine the optimal experimental conditions for TPM imaging, we obtained TPM images of ABI-Nu-labelled HeLa cells under different conditions. The TPM images became increasingly more saturated as laser power, incubation time, and probe concentration were increased (Fig. S9a–h). Therefore, we incubated the cells with 0.5 μM of ABI-Nu for 30 min and used a laser power of 3.68 × 10^9^ mW/cm^2^ at the focal plane to obtain the unsaturated image. The TPM images of the cells co-labelled with with ABI-Nu (0.5 μM) and Pyr-CT (1.0 μM), and with ABI-Nu (0.5 μM) and BF-MT (1.0 μM) were obtained under the same condition (Fig. 3).

Since tissue is thicker than a cell, it took longer to stain the tissues than to stain cells, during which the tissues may have deformed. To minimize this problem, we stained the tissues with excess probe (10 μM). Under such conditions, the probe is expected to exist both in the free and partially aggregated forms. As free probes stained the cells, the equilibrium was shifted, saturating the staining solution with free probes and facilitating staining. We extended the incubation time to 60 min to ensure sufficient staining. We also used a higher laser power (3.80 × 10^9^ mW/cm^2^ at the focal plane) to obtain saturated images of the nucleus to minimize errors in measuring the N-area, the area at the central cross-section enclosed by the nuclear membrane, as unsaturated regions are not recognized by the software (Fig. S9i). The same experimental conditions were used to measure the N/C and M/N ratios.

### Simultaneous detection of DNA with cytoplasm and of DNA with mitochondria in live cells by dual-colour TPM imaging

Using 750 nm TP excitation in scanning lambda mode, HeLa cells labelled with ABI-Nu, Pyr-CT-AM, and BF-MT emitted broad spectra cantered at 473, 561, and 560 nm, respectively (Fig. 3g). Since the emission spectrum of ABI-Nu overlapped appreciably with those of BF-MT and Pyr-CT, we chose 400−475 (Ch1, ABI-Nu) and 600−700 nm (Ch2, Pyr-CT, and BF-MT) as the detection windows (Fig. 3g). Under this condition, the TPEF of Pyr-CT contributed 10% of the total TPEF in Ch1, while the TPEF of ABI-Nu contributed 12% of the total TPEF in Ch2. Similarly, the TPEF of BF-MT contributed 4% of the total TPEF in Ch1, while that of ABI-Nu contributed 8% of the total TPEF in Ch2. These results suggest that the TPEFs of ABI-Nu and Pyr-CT and of ABI-Nu and Pyr-CT in the probe-labelled cells can be detected with minimum interference with each other using Ch1 and Ch2 as the detection windows using dual-colour TPM imaging. Indeed, the TPM image of HeLa cells co-labelled with Pyr-CT and Pyr-CT-AM clearly revealed the nuclei and cytoplasm (Fig. 3a–c), while that co-labelled with ABI-Nu and BF-MT showed the nuclei and mitochondria (Fig. 3d–f). Furthermore, the TPEF intensity in Ch1 decreased dramatically upon digestion with deoxyribonuclease and remained nearly the same upon treatment with ribonuclease (Fig. 3h,i). This outcome again confirmed the high selectivity of ABI-Nu for DNA over RNA.

**Figure 4:**
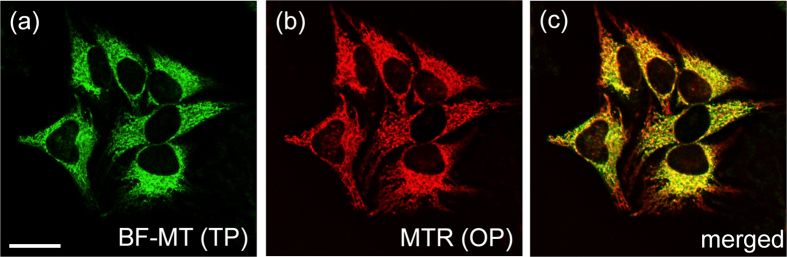
(**a**) TPM and (**b**) OPM images of HeLa cells co-labelled with (**a**) BF-MT (1 μM) and (**b**) Mitotracker Red FM (1 μM) collected with magnification at 100× . (**c**) Co-localized image. The TPEF and OP fluorescence data were collected at 450−600 nm and 650−700 nm upon excitation at 750 nm and 543 nm, respectively. Scale bar, 30 μm. Cells shown are representative images from replicate experiments (n = 5).

To unambiguously confirm that BF-MT localizes in the mitochondria, we performed a co-localization experiment using HeLa cells co-labelled with BF-MT and MitoTracker Red FM (MTR), a commercial one-photon (OP) fluorescence probe for mitochondria^16^, using detection windows of 450–600 nm (BF-MT) and 650–700 nm (MTR), respectively (Fig. S10). The TPM image matched well with the one-photon microscopy (OPM) image of the mitochondria. Pearson’s co-localization coefficient (*A*), which describes the correlation between the intensity distributions of the different channels^22^ of BF-MT with MitoTracker Red FM was 0.85, indicating that BF-MT primarily existed in the mitochondria (Fig. 4a–c).

### Effect of incubation time on the integrity of cells and tissues

Since cells and tissues can become deformed after prolonged incubation, we determined the optimum incubation time during which the N-area and N/C and M/N ratios in the cells and tissues could be measured before deformation began. For this purpose, we incubated HeLa cells and normal colon tissues co-labelled with ABI-Nu and Pry-CT or with ABI-Nu and BF-MT for 0−60 min after staining. The total areas of the nuclei, cytoplasm, and mitochondria in a given sample measured by the TPM images remained nearly the same for 60 min (Fig. S11), indicating that the probe-labelled cells and tissues remained intact for 60 min. Therefore, the N-area and N/C and M/N ratios in the cells and human colon tissues can be measured with minimum interference resulting from deformation (see below).

### Measurements of the N-area and N/C and M/N ratios in human colon tissues

**Figure 5:**
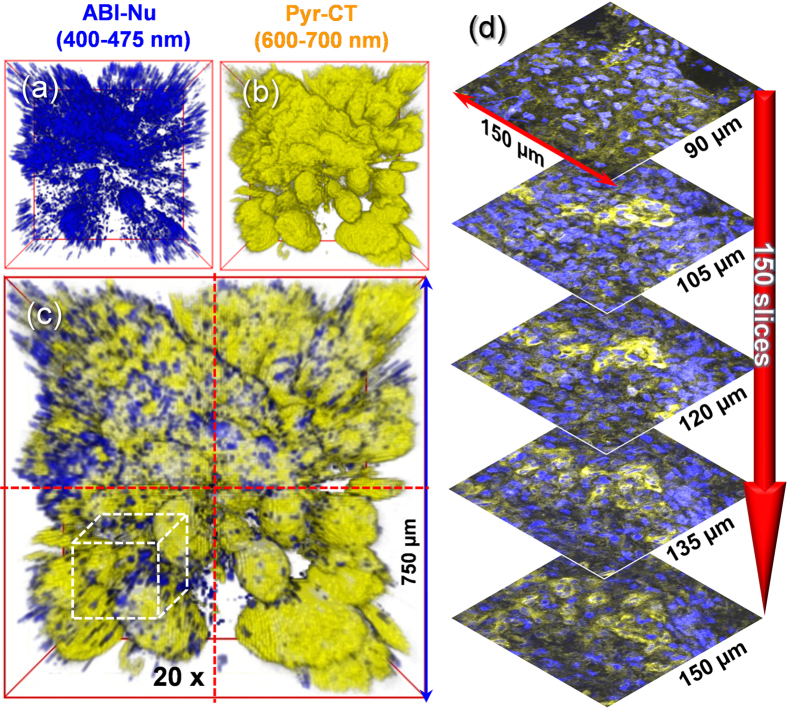
(**a−c**) 3-D TPM image of normal tissue co-labelled with ABI-Nu and Pry-CT collected at (**a**) Ch1 and (**b**) Ch2 at a depth of 90−150 μm at a magnification of 20×. (**c**) Merged image. The dashed red line divides the 4 regions used for detection and the white box indicates the region where 150 sectional images were collected at a magnification of 100×. (**d**) Sectional TPM images of (**c**) obtained at depths of 90−150 μm at a magnification of 100×. Blue and yellow domains represent the nuclei and cytoplasm, respectively. The tissue was incubated with 10 μM probes for 60 min at 37 °C. The TPM images were acquired using 750 nm excitation and the fluorescence emission windows at Ch1 (400−475 nm, blue) and Ch2 (600−700 nm, yellow).

The TPM images of the normal tissues co-labelled with ABI-Nu and Pyr-CT-AM were obtained by collecting the TPEFs in Ch1 (ABI-Nu) and Ch2 (Pyr-CT-AM) in 150 sections at depths of 90−150 μm along the z-direction. The 3-dimensional (3-D) images of the nuclei and cytoplasm were obtained by accumulating the sectional images (Fig. 5a,b), which were then merged to obtain a merged image (Fig. 5c). The 3-D image of the normal tissues co-labelled with ABI-Nu and BF-MT was also obtained using a similar method (Fig. S12).

**Figure 6:**
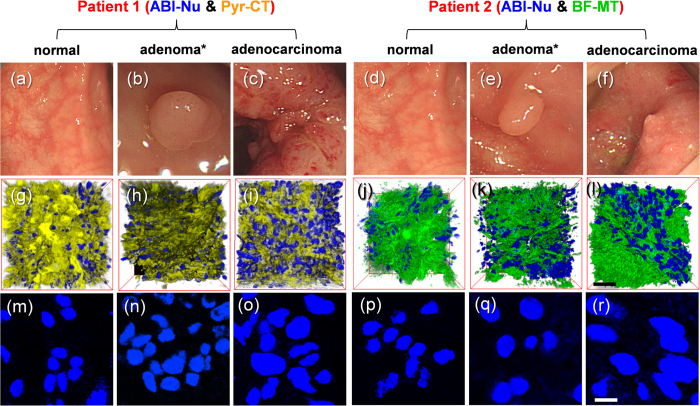
(**a−f**) Colonoscopic images of normal, adenoma (with low grade dysplasia), and adenocarcinoma tissues from patients 1 (**a–c**) and 2 (**d–f**). 3-D images of tissues co-labelled with (**g–i**) ABI-Nu and Pyr-CT, or with (**j–l**) ABI-Nu and BF-MT, at a magnification of 100×. (**m–r**) TPM images of the nuclei in the tissues at a depth of 120 μm at a magnification of 380×. The upper 5% of the regions of fluorescence sorted by decreasing order of area are included. TPM images are shown below the colonoscopic images of the same tissue slice. Blue, yellow, and green domains represent the nuclei, cytoplasm, and mitochondria, respectively. In all experiments, the tissues were incubated with 10 μM probes for 60 min at 37 °C. TPM images were acquired using 750 nm excitation and fluorescence emission windows at Ch1 (400−475 nm, blue) and Ch2 (600−700 nm, yellow). Scale bars, (**l**) 30 and (**r**) 8 μm.

We used 2 tissue slices from the same lesions: normal colon mucosa, tubular adenoma with low grade dysplasia, and moderately differentiated adenocarcinoma tissues to measure the N-area and the N/C and M/N ratios (Fig. 6a–c,d–f). The 3-D images of the tissues co-labelled with ABI-Nu and Pyr-CT-AM (patient 1) revealed the distribution of the nuclei and cytoplasm (Fig. 6g–i), while those co-labelled with ABI-Nu and BF-MT (patient 2) showed the nuclei and mitochondria (Fig. 6j–l). To determine the N-area and N/C and M/N ratios, we obtained 600 TPM images per tissue slice, including 4 TPM images for every 150 sections at a depth of 90−150 μm along the z-direction (Fig. 5c,d).

**Figure 7:**
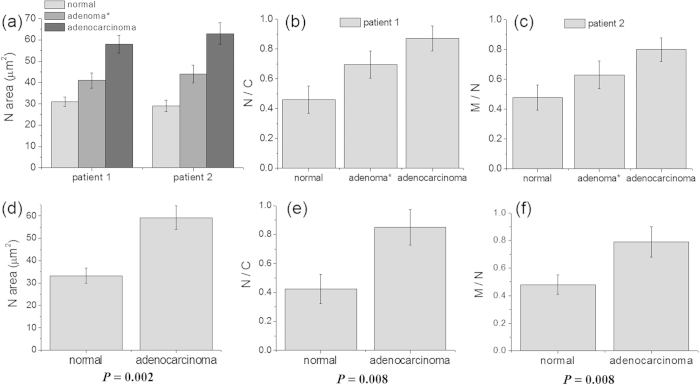
(**a–c**) Average nuclear area (N-area) (**a**), nucleus to cytoplasm (N/C) ratio (**b**), and mitochondria to nucleus (M/N) ratio (**c**) of the normal, adenoma (with low grade dysplasia), and adenocarcinoma tissue slices co-labelled with ABI-Nu and Pyr-CT-AM (**a,b**), or with ABI-Nu and BF-MT (**a,c**). (**d–f**) The N-area (**d**), N/C ratio (**e**), and M/N ratio (**f**) of the normal and adenocarcinoma tissue slices co-labelled with ABI-Nu and Pyr-CT-AM (**d,e**), or with ABI-Nu and BF-MT (**d,f**). (**a–c**) Calculated from 1,200 TPM images (600 × 2 slices) of the tissues from patients 1 and 2, and (**d–f**) 8,400 (600 × 14 slices) (**d**) and 4,200 (600 × 7 slices) (**e,f**) from 7 patients, respectively.

The sectional TPM images obtained at a depth of 120 μm revealed the distribution of the areas of fluorescence, which were sorted in decreasing order (Fig. S13–S15). Since the nuclear area should be at a maximum at the central cross-section, we chose the upper 5% of the areas of fluorescence in the 1200 sectional TPM images (600 images × 2 *ex vivo* slices) and calculated average values to obtain the N-areas. The N-area for patient 1 increased from 31 ± 2 to 41 ± 3 to 58 ± 4 μm^2^, while that for patient 2 increased from 29 ± 3 to 44 ± 4 to 63 ± 5 μm^2^ as the tissue was changed from normal to adenoma to adenocarcinoma (Fig. 7a).

The sectional images also revealed fractions of nuclei, cytoplasm, and mitochondria that belonged to neighbouring cells (Fig. S13–S15). Since a fraction of each nucleus should be accompanied by the cytoplasm and mitochondria that surround it, the N/C and M/N ratios in a given section were calculated by dividing the total areas of fluorescence of the nuclei by those of the cytoplasm and mitochondria using Image-Pro^®^ Analyzer 3D 7.0 software (Media Cybernetics, Rockville, MD, USA). The average N/C and M/N ratios were calculated from 1200 sectional TPM images. The N/C ratio increased from 0.46 ± 0.09 to 0.69 ± 0.10 to 0.87 ± 0.08, while the M/N ratio increased from 0.48 ± 0.08 to 0.63 ± 0.09 to 0.80 ± 0.08 as the tissue became increasingly anomalous (Fig. 7b,c).

We also measured the N-area and the N/C and M/N ratios of 10 additional tissue slices from the normal colon mucosa and moderately differentiated adenocarcinoma (2 tissue slices from the same lesions from 5 patients), respectively, using the method described above. The results for the 7 patients (patients 1 and 2 and 5 others) revealed that the N-area increased significantly from 33.3 ± 3.5 to 59.2 ± 5.3 μm^2^ (P = 0.002), the N/C ratio increased from 0.42 ± 0.09 to 0.86 ± 0.11 (P = 0.008), and the M/N ratio increased from 0.48 ± 0.07 to 0.79 ± 0.11 (P = 0.008) as the normal tissue became cancerous (Fig. 7d–f).

All measurements were completed within 2 h after biopsy, including 60 min for staining, 15 min to obtain the 600 TPM images, and 30 min for data analysis.

## Discussion

A conventional method of detecting colon cancer is pathologic diagnosis using a thin section of paraffin-treated biopsy sample stained with immunohistochemical staining such as hematoxylin and eosin dyes^2^. The cellular morphometry, nuclear size, and N/C ratio are key in pathological diagnosis. An increased nuclear size and high N/C ratio are diagnostic indicators of cytologic atypia in adenocarcinoma^23^,^24^,^25^. However, nuclear size and N/C ratio are subjective findings, not quantifiable values. The discrepancies in these parameters caused by differences in the criteria between east and west as well as inter-observer variations sometimes make it difficult to diagnose early adenocarcinoma and high grade dysplasia/adenoma^3^,^4^. To provide more objective and quantitative information regarding these parameters and a more rapid method of detecting colon cancer, we developed TP probes for DNA (ABI-Nu), cytoplasm (Pry-CT-AM), and mitochondria (BF-MT).

We first studied the binding mode of ABI-Nu with DNA by spectroscopy. The addition of CT-DNA to ABI-Nu caused a red-shift in the λ_max_ and a blue-shift in the λ_fl_ as well as enhanced the *F/F*_*o*_ value (Fig. 2a,b and S1a). This outcome is consistent with the binding of ABI-Nu to CT-DNA, which would flatten the structure and restrict its rotation while providing a more hydrophobic environment, resulting in the observed changes. The *F/F*_*o*_ value was larger for oligonucleotides with a greater number of successive AT sequences and was slightly larger for extended DrewAT than for DrewAT (Fig. 2b and Table S2). In contrast, only slight changes in the *F/F*_*o*_ value were noted in the presence of RNA, misTA, and allCG. These results confirmed that ABI-Nu binds predominantly to double-stranded oligonucleotides containing an AT sequence. Furthermore, most oligonucleotides showed strong positive CD signals at 380 nm in the presence of ABI-Nu; these signals were stronger for DNA containing larger numbers of successive AT sequences (Fig. 2c and S4). Since a strong positive CD signal is observed when a ligand binds to the minor groove of B-DNA, the present result can be attributed to the binding of ABI-Nu to DNA through the AT minor-groove^26^. This outcome is consistent with the similarity in the structures of ABI-Nu and Hoechst 33538, a well-known AT minor-groove binder^27^,^28^.

We next investigated the spectral properties of the probes in a cellular environment. ABI-Nu, Pry-CT, and BF-MT emitted broad TPEF spectra in HeLa cells cantered at 473, 561, and 560 nm with Фδ_eff_ values of 2230, 1555, and 790 GM at 740 nm, respectively (Figs 2d and 3g). The well-separated TPEF spectra and large Фδ_eff_ values allowed simultaneous detection of TPEF signals from ABI-Nu and Pyr-CT and from ABI-Nu and BF-MT in probe-labelled HeLa cells using 400−475 (Ch1, ABI-Nu) and 600−700 nm (Ch2, Pyr-CT, and BF-MT), respectively, as detection windows (Fig. 3g). Furthermore, the TPM image of HeLa cells co-labelled with ABI-Nu and Pyr-CT-AM clearly revealed the nuclei and cytoplasm, while that labelled with ABI-Nu and BF-MT showed the nuclei and mitochondria (Fig. 3a–f). The decrease in the TPEF intensity in the nucleus upon digestion with deoxyribonuclease and minimal change upon treatment with ribonuclease also confirmed the high selectivity of ABI-Nu for DNA over RNA (Fig. 3h,i). In addition, the considerable co-localization between the OPM and TPM images of HeLa cells co-labelled with MTR and BF-MT established the utility of BF-MT as a TP mitotracker (Fig. 4). Additionally, BF-MT emits TPEF at a longer wavelength than does FMT-green (565 vs 540 nm), an existing TP Mitotracker^15^, which is an advantage for dual-colour TPM imaging. Furthermore, the local concentrations of ABI-Nu, Pry-CT, and BF-MT in the respective organelles were 186, 8, and 60 μM, respectively. This outcome is expected considering that ABI-Nu has an isohelical structure with a DNA minor groove and several hydrogen-bonding sites that enhance binding, whereas BF-MT has positive charge that may favourably interact with the negative membrane potential of the mitochondria and favour its localization to the mitochondria. In contrast, the hydrophobic interactions between Pry-CT and the cytoplasm cannot compete with those mentioned above. In particular, ABI-Nu showed higher TP brightness than did Hoechst 33538 (2230 vs 1625 GM) (Fig. 2d and S5), which can be attributed to the larger Фδ_max_ value (10 vs 12 GM) and higher local concentration (160 vs 186 μM). The Фδ_eff_ values can be easily determined and provide a quantitative measure of TP brightness. All probes described showed modest to high photo stability as revealed by the modest to negligible decreases in the TPEF intensities in the probe-labelled HeLa cells, pH-insensitivity at pH 5−7, and minimal cytotoxicity. These results established the utility of these probes for simultaneous detection of the DNA and cytoplasm and of the DNA and mitochondria by dual-colour TPM imaging with minimum interference resulting from photo-decomposition, pH, and cytotoxicity.

We next investigated the use of ABI-Nu, Pyr-CT-AM, and BF-MT for measuring the N-area and N/C and M/N ratios in human colon tissues. The 3-D images of tissues co-labelled with ABI-Nu and Pyr-CT-AM (patient 1) revealed the distribution of the nuclei and cytoplasm (Fig. 6g–i), while those co-labelled with ABI-Nu and BF-MT (patient 2) showed the nuclei and mitochondria (Fig. 6j–l). The distributions of the nuclei cross-sections measured at a 120-μm depth were similar for all tissues; however, the cross-sections were larger for more malignant tissues (Fig. 6m−r). Indeed, the N-area for patient 1 increased from 31 ± 2 to 41 ± 3 to 58 ± 4 μm^2^, while that for patient 2 increased from 29 ± 3 to 44 ± 4 to 63 ± 5 μm^2^ as the tissue was changed from normal to adenoma to adenocarcinoma (Fig. 7a). Also, the N-area calculated for 7 patients increased from 33.3 ± 3.5 to 59.2 ± 5.3 μm^2^ as the tissue was changed from normal to adenocarcinoma (Fig. 7d). The small standard deviations in these values underline the adequacy of the upper 5% criterion used to estimate the N-area. Furthermore, the significantly different N-areas for different lesion categories and markedly similar values for the similarly categorized lesions taken from different patients demonstrate the potential of this method as a diagnostic measure for colon cancer.

The N/C and M/N ratios increased gradually in the order of: normal <adenoma <adenocarcinoma (Fig. 7a–f). For a given type of lesion, the ratios were very similar, likely because the cytoplasm is nearly fully occupied by mitochondria (Fig. 7b,c,e,f). However, these ratios contained large errors that may be attributed to the heterogeneous nature of the *ex vivo* slices and the nature of taking the ratio between two values, each with its own error range. Taken together, measurement of the N-area can be performed with higher accuracy than that of the N/C and M/N ratios and can easily distinguish the differences between various lesion classes with small experimental errors and is similar for the same type of lesion across different patients. Furthermore, the proposed TP method is much faster and more reliable than conventional pathologic diagnosis; all required parameters can be measured from 600 TPM images per slice within 2 h after biopsy using dual-colour TPM imaging. To determine the cut-off values of the N-area and the N/C and M/N ratios for clinical applications, however, studies containing a larger sample size are needed.

## Conclusion

In summary, we developed new TP probes for nuclei (ABI-Nu), cytoplasm (Pyr-CT-AM), and mitochondria (BF-MT) that can simultaneously detect nuclei/cytoplasm and nuclei/mitochondria in human colon tissues by dual-colour TPM imaging. Using these newly developed TP probes, 600 TPM images can be obtained per *ex viv*o slice by dual-colour TPM imaging, from which the N-area and the N/C and M/N ratios can be calculated within 2 h after biopsy. Furthermore, in this study we demonstrated that the N-area and N/C and M/N ratios increased gradually in the order of: normal <adenoma <adenocarcinoma. While the experimental errors in the N/C and M/N ratios were too large to be useful for diagnostic measures, the N-area could readily distinguish the differences between lesion types with small experimental errors and produced similar values for the same type of lesions from different patients, demonstrating its diagnostic potential.

## Methods

Methods and any associated references are available in the online version of the paper.

### Materials

Synthesis of ABI-Nu, Pyr-CT, and BF-M is described in the Supplementary information.

### Spectroscopic measurements

The absorption spectra were recorded using a Hewlett-Packard 8453 diode array spectrophotometer (Palo Alto, CA, USA), and the fluorescence spectra were obtained using an Amico-Bowman series 2 luminescence spectrometer with a 1-cm standard quartz cell. The fluorescence quantum yield was determined using Coumarin 307 and Rhodamine B according to a previously described method^29^. The CD spectra were monitored on a Jasco J-810-150S spectrometer (Easton, MD, USA). Each spectrum was the average of four scans carried out with a 1-nm step from 600 nm to 200 nm. The scanning speed was 200 nm/min. The buffer used in all spectroscopy experiments was 10 mM sodium phosphate buffer, pH 7.4. The spectral data obtained under various conditions are summarized in Fig. S1–4 and Table S1.

### Preparation of nucleic acids

Ext.DrewAT, DrewAT, Ds26, misTA, allAT, and allCG^30^,^31^ were obtained from Integrated DNA Technologies (Coralville, IA, USA) (Table S2). The autocomplementary oligonucleotides (DrewAT, Ext.DrewAT, Ds26, allAT, and allCG) were heated at 95 °C for 5 min in milliQ water and then slowly cooled to room temperature to generate double-stranded DNA structures. Concentrations were determined by measuring UV-Vis absorption using the supplier’s values for the molar extinction coefficients at 260 nm. DNA sodium salt from calf thymus (CT-DNA) and RNA from torula yeast were purchased from Sigma-Aldrich (St. Louis, MO, USA). CT-DNA and RNA were dissolved in 10 mM sodium phosphate buffer (pH 7.4, 100 mM NaCl) and filtered. Concentrations were evaluated by UV considering that an OD 260 nm of 1 corresponds to approximately 50 μg/mL double-stranded DNA and 40 μg/mL RNA, respectively.

### Measurements of TP action cross-section (δФ) and effective TP action cross-section (Фδ_eff_)

The δ values of ABI-Nu, Pyr-CT, and BF-MT in PBS (pH 7.4), 1,4-dioxane/H_2_O (32/1), and EtOH, respectively, were determined using a previously described fluorescence method^20^,^21^. The effective Фδ values (Фδ_eff_) were estimated by comparing the TPEF intensities from the probe-labelled cells and Rhodamine 6G in MeOH (5.0 μM, 1.0 mL) in delta T-dishes in the TPM set-up. The Фδ_eff_ values were calculated by using Фδ_eff_ = Фδ_ref_(*I*_probe_/*I*_ref_), where Фδ_ref_ is the Фδ value of Rhodamine 6G in MeOH, and *I*_probe_ and *I*_ref_ are the TPEF intensities of the probes in each organelle and Rhodamine 6G in MeOH, respectively.

### Cell culture

HeLa human cervical carcinoma cells were obtained from the American Type Culture Collection (ATCC, Manassas, VA, USA). The cells were cultured in DMEM (WelGene Inc., Seoul, Korea) supplemented with heat-inactivated 10% fetal bovine serum (FBS) (WelGene), penicillin (100 U/mL), and streptomycin (100 μg/mL). All cell lines were maintained in a humidified atmosphere of 5% CO_2_ and 95% air at 37 °C. Two days before imaging, the cells were detached and replated on glass-bottom dishes (MatTek, Ashland, MA, USA). For labelling, the growth medium was removed and replaced with DMEM without FBS. The cells were incubated with the probes (2 μM) for 30 min, washed three times with DMEM without FBS, and imaged.

### TPM imaging

Two-photon fluorescence microscope images of probe-labelled HeLa cells and tissues were obtained using spectral confocal and multiphoton microscopes (Leica TCS SP2; Leica Camera, Solms, Germany), using a 100× oil objective with a numerical aperture of 1.30, and a 20× dry objective with a numerical aperture of 0.50. Two-photon excitation was provided using a mode-locked titanium-sapphire laser source (Chameleon, 90 MHz, 200 fs; Coherent Inc., Santa Clara, CA, USA) set at a wavelength and output power of 750 nm and 1264, 1284, and 1305 mW, respectively, which corresponded to a power of (6.06, 6.16, and 6.26) × 10^8^ mW/cm^2^ (×20) and (3.68, 3.74, and 3.80) × 10^9^ mW/cm^2^ (×100) at the focal plane. The TPM images of the HeLa cells were obtained by incubating the cells with ABI-Nu (0.5 μM) and Pyr-CT (1.0 μM), and with ABI-Nu (0.5 μM) and BF-MT (1.0 μM) and by exciting the probes-labeled cells at 750 nm with the laser power of 3.68 × 10^8^ mW/cm^2^ at the focal plane. For the tissue imaging, excess probes (10 μM) and a higher laser power (3.80 × 10^8^ mW/cm^2^ at the focal plane) were used to minimize the errors in the measurements of the N-area, N/C and M/N ratios. Images were obtained in the 400–475 nm (channel 2) and 600–700 nm (channel 2) ranges by using internal PMTs to collect the signals in an 8-bit unsigned 512 × 512 pixel format at a scan speed of 400 Hz.

### Photostability

Photostability was determined under the imaging conditions by monitoring the time-dependent decrease in TPEF intensity in HeLa cells labelled with the studied probes. Measurements were made in four individual cells chosen without bias (Fig. S6).

### pH dependency

The fluorescence intensities of ABI-Nu and Pyr-CT were measured in EtOH/H_2_O (1/1) at different pH values. The probes were pH-insensitive over the range of pH 5.0−8.0 (Fig. S7).

### Cell viability

To confirm that the tracker did not affect the viability of HeLa cells under our incubation conditions, we used a Cell Counting Kit-8 (Dojindo) according to the manufacturer’s protocol. The results are shown in Fig. S8.

### Detection windows

The TPEF spectrum of ABI-Nu in HeLa cells overlapped appreciably with that of Pyr-CT. To minimize the errors due to spectral overlap, we determined the detection windows by considering two factors: i) the TPEF spectra from the two probes should be separated as far as possible and ii) the TPEF intensities from the two probes should be similar. The detection windows meeting the above requirements were 400−475 (Ch1, ABI-Nu) and 600−700 nm (Ch2, Pyr-CT), respectively (Fig. 3g). The detection windows for BF-MT and MTR for the co-localization experiment were 450–600 nm (BF-MT) and 650–700 nm (MTR), respectively (Fig. S10).

### Effect of incubation time on the integrity of cells and tissues

To determine whether the cells and tissue slices remained intact during the prolonged incubation, we obtained TPM images of HeLa cells and normal colon tissues co-labelled with ABI-Nu and Pyr-CT and with ABI-Nu and BF-MT after incubation for 0−60 min. The total areas of the nuclei and cytoplasm in the probe-labelled cells and tissues in the 150 × 150 μm panels were measured using the cell analysis program in Gen5^TM^ software (BioTek, Winooski, VT, USA), which automatically calculates the areas of the fluorescent regions. Since the areas differed depending on the samples, the values measured in each sample at t = 0 were used as a reference and thus were given the value 1.0. The total areas measured at different times from the TPM images of 10 different cell samples and 30 sectional TPM images of the tissue slices obtained at depths of 90−150 μm were averaged. They remained nearly the same within the range of experimental error for 60 min (Fig. S11).

### Measurements of the N-areas and the N/C and M/N ratios in human colon tissues

*Ex vivo* colon slices were obtained with informed consent from 7 outpatients who had undergone elective colonoscopies at Korea University Medical Center, Anam Hospital, in accordance with the approved institutional review board protocol. We used two tissue slices from the same lesions: normal colon mucosa, tubular adenoma with low grade dysplasia, and moderately differentiated adenocarcinoma tissues, as diagnosed by a pathologist (Fig. 6a–c,j–l), for the measurements. The tissues from patient 1 were used to determine the N-areas and N/C ratios, while those from patient 2 were used to determine the N-areas and M/N ratios. We used excess amounts of ABI-Nu (10 μM), Pyr-CT-AM (10 μM), and BF-MT (10 μM) to facilitate staining, and the tissues were incubated with the probes for 60 min to ensure sufficient staining. We obtained 600 TPM images per *ex vivo* slice co-labelled with ABI-Nu and Pyr-CT, and with ABI-Nu and BF-MT, consisting of 4 TPM images at every 150 sections at depths of 90−150 μm along the z-direction. The TPM images were collected at Ch1 (ABI-Nu) and Ch2 (Pyr-CT-AM, BF-MT) at a magnification of 100×. The sectional TPM images obtained at the 120-μm depth revealed the distribution of the fluorescence areas. Count analysis was performed using Image-Pro^®^ Analyzer 3D 7.0 software (Media Cybernetics) to sort the areas of fluorescence in decreasing order (Fig. S13–S15). Since the nuclear area should be maximized at the central cross-section, we selected the upper 5% of the areas of fluorescence in 1200 sectional TPM images (600 images × 2 *ex vivo* slices) and calculated average values to obtain the N-areas. The sectional images also revealed fractions of the nuclei, cytoplasm, and mitochondria belonging to neighbouring cells (Fig. S13–S15). Since a nuclear fraction should be accompanied by the cytoplasm and mitochondria belonging to the same nucleus, the N/C and M/N ratios in a given section were calculated by dividing the total areas of fluorescence of the nuclei by those of the cytoplasm and mitochondria. The average N-area and the N/C and M/N ratios were also calculated from 2400 (600 × 4 tissue slices) and 1200 (600 × 2 tissue slices) sectional TPM images, respectively.

We also measured the N-area and N/C and M/N ratios of the normal colon mucosa and moderately differentiated adenocarcinoma tissues obtained from 5 other patients using the method described above. Again, two tissue slices from the same lesions were used in the measurements. The results were combined with those obtained from patients 1 and 2, that is, the average N-area and the N/C and M/N ratios of the normal colon mucosa and moderately differentiated adenocarcinoma tissues were calculated from 8,400 (600 × 14 tissue slices) and 4,200 (600 × 17 tissue slices) sectional TPM images, respectively.

### Statistical analysis

This was a pilot study. The significance of differences in N area, N/C ratio, and M/N ratios in normal and adenocarcinoma tissues were analyzed using the Wilcoxon signed rank test. The results are described as the mean ± SD. The significance level was set at P < 0.05.

## Additional Information

**How to cite this article**: Lim, C. S. *et al*. Measurement of the Nucleus Area and Nucleus/Cytoplasm and Mitochondria/Nucleus Ratios in Human Colon Tissues by Dual-Colour Two-Photon Microscopy Imaging. *Sci. Rep.* **5**, 18521; doi: 10.1038/srep18521 (2015).

## Supplementary Material

Supplementary Information

## Figures and Tables

**Figure 1:**
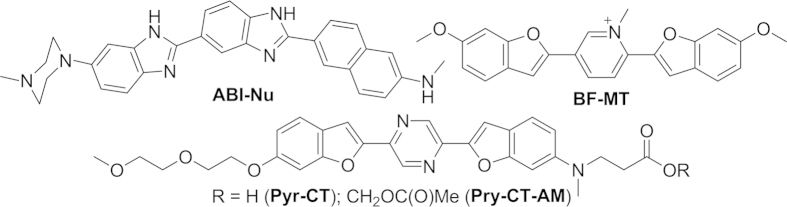
Structures of ABI-Nu, BF-MT, Pry-CT, and Pry-CT-AM.
