# Supplementary material for: Measurement of the Nucleus Area and Nucleus/Cytoplasm and Mitochondria/Nucleus Ratios in Human Colon Tissues by Dual-Colour Two-Photon Microscopy Imaging
Source: Sci Rep. 2015 Dec 17;5:18521. doi: 10.1038/srep18521 (PMC4682082; doi:10.1038/srep18521)
Supplement: Supplementary Information [file srep18521-s1.doc]

Supporting Information to Accompany “**Measurement of the Nucleus Area and Nucleus/Cytoplasm and Mitochondria/Nucleus Ratios in Human Colon Tissues by Dual-Colour Two-Photon Microscopy Imaging**”

Chang Su Lim, Eun Sun Kim, Ji Yeon Kim, Seung Taek Hong, Hoon Jai Chun,* Dong Eun Kang,* and Bong Rae Cho*

*Department of Chemistry, Korea University, 145 Anam-ro, Sungbuk-gu, Seoul 136-713, Korea. Fax: +82-2-3290-3121; E-mail:* [*chobr@korea.ac.kr*](mailto:chobr@korea.ac.kr)*, dongeun@korea.ac.kr; Department of Internal Medicine, Korea University College of Medicine, 73 Inchon-ro, Seoul, 136-705, Korea. E-mail: drchunhj@chol.com; KU-KIST Graduate School of Converging Science and Technology, Korea University, 145 Anam-ro, Sungbuk-gu, Seoul 136-713, Korea*

| Table of Contents | Page |
| --- | --- |
| Synthesis of **ABI-Nu**, **BF-MT,** and **Pyr-CT-AM**………………………………………………………. | S3 |
| Solubility of **ABI-Nu**, **BF-MT,** and **Pyr-CT-AM** in phosphate buffer.………………………………… | S6 |
| Reference…………………………………………………………………………………………………. | S26 |
| **Figure S1**. (a–c) Normalized absorption and (d–f) emission spectra of (a, d) **ABI-Nu** in PBS buffer in the absence and presence of CT-DNA and allAT, (b, e) **Pyr-CT-AM** in EtOH and dioxane/H_2_O (32/1), and (c, f) **BF-MT** in PBS buffer and EtOH**.** ……………………………………………………………… | S6 |
| **Figure S2**. (a, c) One-photon fluorescence and (b) absorption spectra. Plot of (d, f) fluorescence and (e) absorption intensity against dye concentration for **ABI-Nu**, **Pyr-CT-AM**, and **BF-MT** in phosphate buffer. The excitation wavelength was 360 nm (a, c). | S7 |
| **Figure S3**. Fluorimetric titration curves obtained by plotting the fluorescence enhancement *F*/*F*_0_ vs the concentrations of CT-DNA and RNA. | S8 |
| **Figure S4**. CD spectra of allCG (a), Ext.DrewAT (b), DrewAT (c), and Ds26 (d) in the presence of ABI-Nu (0–64 µM) in 10 mM PBS pH 7.4. In all cases, the oligonucleotide concentration was 10 µM. | S8 |
| **Figure S5**. Two-photon action spectra of (a) **ABI-Nu** and Hoechst 33538 in PBS in the absence (-■-) and presence (-○-) of allAT, (b) **Pyr-CT** in 1,4-dioxane/H_2_O (32/1), and (c) **BF-MT** in EtOH.  **Figure S6**. TPM images of HeLa cells labelled with **ABI-Nu** (a), **Pyr-CT** (b), and **BF-MT** (c). The cells were incubated with 1 μM each of **ABI-Nu**, **Pyr-CT,** and **BF-MT** for 30 min at 37°C. (b, e, f) Relative TPEF intensities measured at A–C in the upper column as a function of time. The TPEFs were collected at 400–650 nm upon excitation at 750 nm with fs pulse. | S9  S9 |
| **Figure S7**. Effect of pH on the one-photon fluorescence intensity of **ABI-Nu** (a), **Pyr-CT** (b), and **BF-MT** (c) in PBS. The excitation wavelength was 365 nm. | S9 |
| **Figure S8**. Viability of HeLa cells in the presence of **ABI-Nu** (a), **Pyr-CT** (b), and **BF-MT** (c) as measured using a CCK-8 kit. The cells were incubated with 1–10 μM probes for 2–6 h. | S10 |
| **Figure S9**. TPM images of HeLa cells labelled with **ABI-Nu** obtained by varying (a–c) laser power, (d–f) incubation time, and (g–i) probe concentration. Unless otherwise noted, the laser power at the focal point, incubation time, and probe concentration were 3.80 × 10^9^ mW/cm^2^, 30 min, and 1.0 μM, respectively………………………………………………………………………………………………  **Figure S10**. One-photon fluorescence spectra of MTR and two-photon excited fluorescence spectra of **BF-MT** in HeLa cells. The one- and two-photon excitation wavelengths were 543 (MTR) and 750 (**BF-MT**), respectively. ……………………………………………………………………………………….  **Figure S11**. TPM images of HeLa cells (a, g) and human colon tissues (d, j) co-labelled with **ABI-Nu** and **Pyr-CT** (a, d), and **ABI-Nu** and **BF-MT** (g, j) obtained at 0 and 60 min after staining. The total areas of the nuclei (b, e, h, k), cytoplasm (c, f), and mitochondria (i, l) in HeLa cells and human colon tissue were measured after incubation for 0−60 min. Cells and tissues shown are representative images from replicate experiments (n = 5). The TPEFs were collected at 400–475 nm (Ch1, **ABI-Nu**) and 600–700 nm (Ch2, **Pyr-CT** and **BF-MT**) upon excitation at 750 nm………………………………………..  **Figure S12**. 3-D TPM images of normal colon tissue co-labelled with ABI-Nu and BF-MT collected in (a) Ch1 and (b) Ch2 at the depth of 90–150 μm with magnification at 20×. (c) Merged image. (d) Sectional TPM images obtained at the depth of 90−150 μm with magnification at 100×. Blue and yellow dots represent the nuclei and mitochondria, respectively………………………………………………..  **Figure S13.** (a, d, g) Sectional TPM image of the (a) normal, (d) adenoma (with low-grade dysplasia), and (g) adenocarcinoma co-labelled with **ABI-Nu** and **Pyr-CT** collected at Ch1 at a depth of 120 μm. (b, e, h) Sorted images in decreasing order of area. Count analysis was performed using Image-Pro® Analyzer 3D 7.0 software (Media Cybernetics). White marks in a, d, and g indicate the fluorescent regions identified by the software. (b, e, h) Dotted whited boxes include the fluorescent regions whose TPEF intensities were 90% of the maximum. (c, f, i) Bar diagram showing the distribution of the nuclei in decreasing order of area in b, e, and h. The vertical lines indicate regions where the TPEF intensity was 90% of the maximum……………………………………………………………………………….  **Figure S14.** (a, d, g) Sectional TPM image of the (a) normal, (d) adenoma (with low grade dysplasia), and (g) adenocarcinoma co-labelled with **ABI-Nu** and **Pyr-CT** collected at Ch2 at a depth of 120 μm. (b, e, h) Sorted images in decreasing order of area. Count analysis was performed using Image-Pro® Analyzer 3D 7.0 software (Media Cybernetics). White marks in a, d, and g indicate the fluorescent regions recognized by the software. (b, e, h) Dotted whited boxes include the fluorescent regions whose TPEF intensities were 90% of the maximum. (c, f, i) Bar diagram showing the distribution of the nuclei in decreasing order of area in b, e, and h. The vertical lines indicate regions where the TPEF intensity was 90% of the maximum………………………………………………………………………………..  **Figure S15.** (a, d, g) Sectional TPM image of the (a) normal, (d) adenoma (with low-grade dysplasia), and (g) adenocarcinoma co-labelled with **ABI-Nu** and **BF-MT** collected at Ch2 at a depth of 120 μm. (b, e, h) Sorted images in decreasing order of area. Count analysis was performed using Image-Pro® Analyzer 3D 7.0 software (Media Cybernetics). White marks in a, d, and g indicate the fluorescent regions identified by the software. (b, e, h) Dotted whited boxes include the fluorescent regions whose TPEF intensity was 90% of the maximum. (c, f, i) Bar diagram showing the distribution of the nuclei in decreasing order of area in b, e, and h. The vertical lines indicate the regions where the TPEF intensity was 90% of the maximum…………………………………………………………………………………  **Figure S16-S35**. ^1^H, ^13^C NMR, and HRMS spectra of **3**-**20**, **ABI-Nu**, **BF-MT,** and **Pyr-CT-AM**……… | S10  S11  S12  S13  S14  S15  S16  S17 |
| **Table S1**. Absorption and emission maxima of **ABI-Nu**, **BF-MT**, and **Pyr-CT-AM** in various solvents. | S6 |
| **Table S2**. Sequences and structures of the oligonucleotides used in this study. | S7 |

**Syntheses of ABI-Nu**, **Pyr-CT-AM**, and **BF-MT**. Compounds **1**, **2**, **5**, **6**, and **7**^1-5^ were prepared using published methods. Syntheses of **ABI-Nu**, **Pyr-CT-AM**, and **BF-MT** are described below.

**Scheme S1**. (a) Na_2_S_2_O_5_, EtOH, H_2_O; (b) H_2_, Pd/C, AcOH.

**Scheme S2**. a) Bromomethyl acetate, TEA, DMF, rt.

**Scheme S3**. (a) 4-Methoxysalicylaldehyde, K_2_CO_3_, DMF, 90°C; (b) LAH, THF, 0°C; (c) DDQ, PPh_3_, Bu_4_NBr, CH_2_Cl_2_, rt; (d) 4-methoxysalicylaldehyde, K_2_CO_3_, DMF, reflux; (e) MeI, reflux.

**Compound 3**. A solution of Na_2_S_2_O_5_ (170 mg, 0.89 mmol, 1.5 equiv) in 0.2 mL water was added to a solution containing **1** (110 mg, 0.58 mmol) and **2** (120 mg, 0.58 mmol, 1 equiv) in EtOH (2 mL). The mixture was refluxed overnight and cooled to room temperature. The solvent was removed *in vacuo* and the residue was subjected to flash column chromatography using 5% MeOH/CHCl_3_ as the eluent to afford 95 mg (43%) of **3** as a brown solid: ^1^H NMR (300 MHz, acetone-*d*_6_); δ 8.78 (1H, s), 8.63 (1H, d, *J* = 8.4 Hz), 8.30 (1H, d, *J =* 8.4 Hz), 7.57 (1H, d, *J =* 8.4 Hz), 7.08–7.11 (2H, m), 3.26 (4H, t, *J* = 4.2 Hz), 2.69 (4H, t, *J* = 4.2 Hz), 2.39 (3H, s).

**Compound 4**. A suspension of **3** (90 mg, 0.23 mmol) and 5% Pd/C catalyst in AcOH was stirred under H_2_ for 2 h at room temperature. The catalyst was filtered off and the filtrate was concentrated *in vacuo* to afford 78 mg (95%) of **4** as a brown solid: ^1^H NMR (300 MHz, DMSO-*d*_6_); *δ* 7.51 (1H, d, *J* = 1.8 Hz), 7.31–7.37 (2H, m), 7.00 (1H, d, *J =* 1.8 Hz), 6.86 (1H, dd, *J =* 8.4, 1.8 Hz), 6.70 (1H, d, *J =* 8.4 Hz), 3.12 (4H, t, *J* = 4.2 Hz), 2.52 (4H, t, *J* = 4.2 Hz), 2.26 (3H, s). ^13^C NMR (100 MHz, DMSO-*d*_6_); *δ* 172.7, 159.6, 159.3, 149.5, 148.6, 136.0, 134.9, 125.9, 120.6, 115.6, 105.4, 101.1, 55.1, 49.9, 45.3.

**ABI-Nu**. A solution of Na_2_S_2_O_5_ (17 mg, 0.090 mmol, 1.5 equiv) in 0.1 mL H_2_O was added to a solution containing **4** (20 mg, 0.060 mmol) and **5** (13 mg, 0.074 mmol, 1.2 equiv) in EtOH (1 mL). The mixture was refluxed overnight and cooled to room temperature. The solvent was removed *in vacuo* and the residue was subjected to flash column chromatography using MeOH as the eluent to afford 20 mg (66%) of **ABI-Nu** as a yellow solid: ^1^H NMR (300 MHz, CD_3_OD); *δ* 8.40 (1H, s), 8.28 (1H, s), 8.05 (1H, dd, *J* = 8.4, 1.8 Hz), 7.96 (1H, dd, *J* = 8.4, 1.8 Hz), 7.75–7.71 (3H, m), 7.52 (1H, d, *J* = 8.4 Hz), 7.16 (1H, d, *J* = 1.8 Hz), 7.07-–7.00 (2H, m), 6.79 (1H, d, *J* = 1.8 Hz), 3.27 (4H, t, *J* = 4.2 Hz), 2.91 (3H, s), 2.81 (4H, t, *J* = 4.2 Hz), 2.48 (3H, s). ^13^C NMR (100 MHz, CD_3_OD); *δ* 154.8, 149.5, 148.3, 137.0, 129.3, 126.7, 126.6, 126.3, 124.3, 123.7, 121.6, 121.2, 118.8, 115.1, 102.2, 54.9, 50.5, 44.9, 29.1. HRMS (FAB^+^): m/z calculated for [C_30_H_29_N_7_+H^+^]: 488.2518, observed: 488.2564.

**Pyr-CT-AM**. A mixture of **6** (50 mg, 0.094 mmol), bromomethyl acetate (0.14 g, 0.94 mmol), and triethylamine (0.14 g, 1.4 mmol) in DMF (3 mL) was stirred under Ar overnight. The solvent was removed *in vacuo* and the crude product was purified by column chromatography using ethyl acetate/hexane (2:1) as the eluent. Yield: 29 mg (51%); ^1^H NMR (500 MHz, CDCl_3_): δ 9.02 (2H, s), 7.54–7.49 (2H, m), 7.43 (1H, s), 7.42 (1H, s), 7.14 (1H, d, *J* = 2.2 Hz), 6.96 (1H, dd, *J* = 8.8, 2.2 Hz), 6.94 (1H, d, *J* = 2.2 Hz), 6.82 (1H, dd, *J* = 8.8, 2.2 Hz), 5.76 (2H, s), 4.25–4.21 (2H, m), 3.95–3.91 (2H, m), 3.82-3.75 (4H, m), 3.64–3.60 (2H, m), 3.42 (3H, s), 3.06 (3H, s), 2.74–2.69 (2H, m), 2.11 (3H, s). ^13^C NMR (100 MHz, CDCl_3_): δ 171.3, 170.9, 158.3, 157.8, 156.6, 152.1, 150.7, 148.2, 143.1, 142.2, 140.3, 140.1, 122.1, 122.0, 121.9, 118.9, 113.5, 110.7, 107.0, 106.3, 96.7, 94.7, 79.2, 71.9, 70.8, 69.7, 67.9, 59.1, 48.7, 38.8, 31.5, 20.7 ppm; HRMS(FAB^+^): m/z calculated for [C_32_H_33_N_3_O_9_+H^+^]: 604.2290, observed: 604.2289.

**Compound 8**. A mixture of **7** (200 mg, 0.87 mmol), 4-methoxysalicylaldehyde (130 mg, 0.87 mmol) and K_2_CO_3_ (360 mg, 2.6 mmol) was stirred in DMF for 8 h at 90°C. The dark reaction mixture was diluted with water and extracted with CH_2_Cl_2_. The organic layer was evaporated and the residue was subjected to silica gel column chromatography using CH_2_Cl_2_/EtOAc (4/1) as the eluent to afford **8** as a yellow solid (180 mg, 75%). ^1^H NMR (300 MHz, CDCl_3_); *δ* 9.23 (1H, d, *J* = 2.2 Hz), 8.35 (1H, dd, *J* = 8.5, 2.2 Hz), 7.88 (1H, d, *J* = 8.5 Hz), 7.53 (1H, d, *J* = 8.5 Hz), 7.50 (1H, s), 7.10 (1H, d, *J* = 2.2 Hz), 6.93 (1H, dd, *J* = 8.5, 2.2 Hz), 3.98 (3H, s), 3.89 (3H, s). ^13^C NMR (100 MHz, CDCl_3_); *δ* 165.8, 159.6, 157.1, 153.7, 152.7, 151.4 138.1, 124.3, 122.5, 122.1, 118.7, 113.2, 107.5, 96.0, 55.9, 52.6.

**Compound 9**. LiAlH_4_ (0.26 mL, 0.52 mmol, 2.0 M in THF) was added to a solution of **8** (100 mg, 0.35 mmol) in THF (5 mL) at 0°C and the mixture was stirred for 30 min. The reaction mixture was quenched with a small amount of water and extracted with EtOAc. The organic layer was evaporated and the residue was subjected to silica gel column chromatography using CH_2_Cl_2_/EtOAc (1/1) as the eluent to afford **9** as a yellow product (71 mg, 80%). ^1^H NMR (300 MHz, CDCl_3_); *δ* 8.60 (1H, d, *J* = 2.2 Hz), 7.78–7.82 (2H, m), 7.50 (1H, d, *J* = 8.5 Hz), 7.34 (1H, s), 7.09 (1H, d, *J* = 2.2 Hz), 6.90 (1H, dd, *J* = 8.5, 2.2 Hz), 4.76 (2H, s), 3.87 (3H, s). ^13^C NMR (100 MHz, CDCl_3_); *δ* 159.0, 156.6, 154.3, 149.0, 148.8, 135.9, 135.1, 122.3, 122.0, 119.4, 112.7, 105.0, 96.1, 62.9, 55.9.

**Compound 10**. Bu_4_N^+^Br^-^ (225 mg, 0.70 mmol) was added to a mixture containing PPh_3_ (183 mg, 0.70 mmol) and 2,3-dichloro-5,6-dicyanobenzoquinone (159 mg, 0.70 mmol) in CH_2_Cl_2_ (10 mL) and stirred for 10 min at room temperature. **9** (100 mg, 0.35 mmol) was then added to the mixture and stirred for 30 min at room temperature. The reaction was quenched with water and the product was extracted with CH_2_Cl_2_. The organic layer was dried over Na_2_SO_4_, filtered, and concentrated. The residue was subjected to silica gel column chromatography using CH_2_Cl_2_/EtOAc (5/1) as the eluent to afford **10** (72 mg, 65%). ^1^H NMR (300 MHz, CDCl_3_); *δ* 8.64 (1H, s), 7.76–7.81 (2H, m), 7.51 (1H, d, *J* = 8.5 Hz), 7.37 (1H, s), 7.08 (1H, d, *J* = 2.2 Hz), 6.90 (1H, dd, *J* = 8.5, 2.2 Hz), 4.51 (2H, s), 3.87 (3H, s). ^13^C NMR (100 MHz, CDCl_3_); *δ* 159.1, 156.7, 154.1, 150.1, 149.3, 137.5, 132.4, 122.3, 122.2, 119.4, 112.9, 105.7, 96.1, 55.9, 30.1.

**Compound 11**. A mixture of **10** (50 mg, 0.13 mmol), 4-methoxysalicylaldehyde (21 mg, 0.13 mmol), and K_2_CO_3_ (75 mg, 0.54 mmol) in 3 mL DMF was refluxed overnight. The dark reaction mixture was diluted with water and extracted with CH_2_Cl_2_. The organic solvent was evaporated and the residue was subjected to silica gel column chromatography using CH_2_Cl_2_/EtOAc (3/1) as the eluent to afford **11** as a yellow solid (40 mg, 63%). ^1^H NMR (300 MHz, CDCl_3_); *δ* 9.08 (1H, d, *J* = 2.2 Hz), 8.13 (1H, dd, *J* = 8.5, 2.2 Hz), 7.88 (1H, d, *J* = 8.5 Hz), 7.53 (1H, d, *J* = 8.5 Hz), 7.48 (1H, d, *J* = 8.5 Hz), 7.39 (1H, s), 7.11–7.09 (2H, m), 7.08 (1H, s), 6.94–6.88 (2H, m), 3.89 (6H, s). ^13^C NMR (100 MHz, CDCl_3_); *δ* 159.1, 158.8, 156.7, 156.4, 154.4, 152.3, 148.3, 146.2, 132.0, 125.5, 122.4, 122.3, 122.1, 121.5, 119.3, 112.8, 112.7, 105.4, 102.9, 96.1, 96.0, 56.0, 55.9.

**BF-MT**. Compound **11** (20 mg, 0.054 mmol) in MeI (3 mL) was refluxed for 1 h. The mixture was cooled in an ice bath and filtered with CH_2_Cl_2_ to afford **BF-MT** as a yellow powder (18 mg, 86%). ^1^H NMR (300 MHz, DMSO-*d*_6_); *δ* 9.69 (1H, s), 8.91 (1H, d, *J* = 8.5 Hz), 8.58 (1H, d, *J* = 8.5 Hz), 8.17 (1H, s), 7.90 (1H, s), 7.79 (1H, d, *J* = 8.5 Hz), 7.73 (1 H, d, *J* = 8.5 Hz), 7.40 (1 H, d, *J* = 2.2 Hz), 7.28 (1 H, d, *J* = 2.2 Hz), 7.07 (1H, dd, *J* = 8.5, 2.2 Hz), 7.03 (1H, dd, *J* = 8.5, 2.2 Hz), 4.61 (3H, s) 3.88 (3H, s), 3.86(3H, s). ^13^C NMR (100 MHz, DMSO-*d*6); *δ* 161.5, 160.0, 159.3, 157.3, 156.9, 148.4, 146.3, 144.4, 143.5, 142.5, 138.6, 127.8, 127.4, 124.3, 123.3, 122.8, 122.3, 121.9, 121.7, 119.7, 117.3, 56.4, 56.3, 49.3. HRMS (FAB^+^): m/z calculated for [C_24_H_20_NO_4_^+^]: 386.1392, observed: 386.1391.

**Figure S1**. (a–c) Normalized absorption and (d–f) emission spectra of (a, d) **ABI-Nu** in PBS buffer in the absence and presence of CT-DNA and allAT, (b, e) **Pyr-CT-AM** in EtOH and dioxane/H_2_O (32/1), and (c, f) **BF-MT** in PBS buffer and EtOH.

**Table S1**. Absorption and emission maxima of **ABI-Nu, Pyr-CT**, and **BF-MT** in various solvents.

| Solvent | **ABI-Nu** | | | **Pyr-CT** | | | **BF-MT** | | |
| --- | --- | --- | --- | --- | --- | --- | --- | --- | --- |
|  | 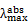/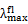^[a]^ | Ф^[b]^ | Фδ_max_^[c]^ | 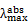/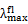^[a]^ | Ф^[b]^ | Фδ_max_^[c]^ | 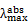/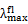^[a]^ | Ф^[b]^ | Фδ_max_^[c]^ |
| HeLa Cell | - /473 | - | - | -/561 | - | - | - /560 | - | - |
| 1,4-Dioxane |  |  | - | 442/530 | 0.91 | - | 374/538 | 0.046 | - |
| 1,4-Dioxane  :H_2_O (32:1) | - | - | - | 442/560 | 0.43 | 194 | - | - | - |
| EtOH | 363/452 | 0.45 | - | 457/577 | 0.020 | - | 374/550 | 0.040 | 13 |
| Buffer^[d]^ | 362/472 | 0.0043 | - | 437/- | - | - | 420/- | - | - |
| CT-DNA^[e]^ | 374/463 | 0.67 | 8.7 | - | - | - | - | - | - |
| allAT^[f]^ | 376/463 | 0.94 | 12 | - | - | - | - | - | - |

^[a]^λ_max_ of the one-photon absorption and emission spectra in nm. ^[b]^Fluorescence quantum yield. The uncertainty is ± 15%. ^[c]^Two-photon action cross section in 10^-50^ cm^4^s/photon (GM), ± 15%. ^[d]^ PBS buffer (pH 7.2); the$E_{\text{T}}^{\text{N}}$value is for water. ^[e]^Deoxyribonucleic acid sodium salt from calf thymus. ^[f]^Autocomplementary oligonucleotides.

**Solubility in phosphate buffer.** A small amount of dye was dissolved in DMSO to prepare the stock solutions (5.0 × 10^-3^ M). The solution was diluted to 1.0 × 10^-3^ M and added to a cuvette containing 3.0 mL PBS (phosphate buffer, pH = 7.4) using a micro syringe. The solution was diluted to (6.0 × 10^-3^–6.0 × 10^-5^) M and added to a cuvette containing 3.0 mL PBS using a micro syringe. In all cases, the concentration of DMSO in PBS was maintained at 0.2 %.^6^ The plots of absorbance and fluorescence intensity against the dye concentration were linear at low concentrations and showed downward curvature at higher concentrations (Figure S1). The maximum concentration in the linear region was taken as the solubility. The solubilities of **ABI-Nu**, **Pyr-CT-AM**, and **BF-MT** in buffer were ~3.0, 4.0, and 6.0 μM, respectively.

**Figure S2**. One-photon fluorescence spectra of **ABI-Nu** (a) and **BF-MT** (c), and absorption spectrum of **Pyr-CT-AM** (b) in phosphate buffer. Plot of (d, f) fluorescence and (e) absorption intensity against dye concentration for **ABI-Nu**, **Pyr-CT-AM**, and **BF-MT** in phosphate buffer, respectively. The excitation wavelength was 360 nm (a, c).

**Table S2**. Sequence and structure of the oligonucleotides used in this study.

| Oligonucleotides | Sequence (5′ → 3′) | Structure |
| --- | --- | --- |
| allAT | AATAAATTTATT | B-DNA |
| Ext.DrewAT | CAATCGCGCGAAATTTCGCGCGATTG | B-DNA |
| DrewAT | CGCGAAATTTCGCG | B-DNA |
| Ds26 | CAATCGGATCGAATTCGATCCGATTG | B-DNA |
| misTA | GGGTTACTACGAACTGG | Single strand |
| allCG | CCGGCGCGCCGG | B-DNA |

**Figure S3**. Plots of the fluorescence enhancement factor (*F*/*F*_0_ ) vs oligonucleotide concentration.

**Figure S4**. CD spectra of allCG (a), Ext.DrewAT (b), DrewAT (c), and Ds26 (d) in the presence of ABI-Nu (0-64 µM) in 10 mM PBS buffer pH 7.4. In all cases, the oligonucleotide concentration was 10 µM.

**Figure S5**. Two-photon action spectra of (a) **ABI-Nu** and Hoechst 33538 in PBS buffer in the absence (-■-) and presence (-○-) of allAT, (b) **Pyr-CT** in 1,4-dioxane/H_2_O (32/1), and (c) **BF-MT** in EtOH.

**Figure S6**. TPM image of HeLa cells labelled with **ABI-Nu** (a), **Pyr-CT** (b), and **BF-MT** (c). The cells were incubated with 1.0 μM each of **ABI-Nu**, **Pyr-CT** and **BF-MT** for 30 min at 37°C. (b, e, f) Relative TPEF intensity measured at A–C in the upper column as a function of time. The TPEF was collected at 400–650 nm upon excitation at 750 nm with fs pulse.

**Figure S7**. Effect of pH on the one-photon fluorescence intensity of **ABI-Nu** (a), **Pyr-CT** (b), and **BF-MT** (c) in EtOH/H_2_O. The excitation wavelength was 365 nm.

**Figure S8**. Viability of HeLa cells in the presence of **ABI-Nu** (a), **Pyr-CT** (b), and **BF-MT** (c) as measured by using CCK-8 kit. The cells were incubated with 1–10 μM of probes for 2–6 h.

**Figure S9**. TPM images of HeLa cells labelled with **ABI-Nu** obtained by varying (a–c) laser power, (d–f) incubation time, and (g–i) probe concentration. Unless otherwise noted, the laser power at the focal point, incubation time, and probe concentration were 3.80 × 10^9^ mW/cm^2^, 30 min, and 1.0 μM, respectively.

**Figure S10**. One-photon fluorescence spectra of MTR and two-photon excited fluorescence spectra of **BF-MT** in HeLa cells. The one- and two-photon excitation wavelengths were 543 (MTR) and 750 (**BF-MT**), respectively.

**Figure S11**. TPM images of HeLa cells (a, g) and normal colon tissues (d, j) co-labelled with **ABI-Nu** and **Pyr-CT** (a, d), and **ABI-Nu** and **BF-MT** (g, j) obtained at 0 and 60 min after staining. Total area of the nucleus (b, e, h, k), cytoplasm (c, f), and mitochondria (i, l) in the probe-labelled HeLa cells and normal colon tissue measured at 0, 15, 30, 45, and 60 min after incubation. The images are the representative images from replicate experiments (n = 5). The TPEF was collected at 400–475 nm (Ch1, **ABI-Nu**) and 600–700 nm (Ch2, **Pyr-CT** and **BF-MT**) upon excitation at 750 nm.

**Figure S12.** 3-D TPM image of normal colon tissue co-labelled with ABI-Nu and BF-MT collected at (a) Ch1 and (b) Ch2 at depths of 90–150 μm at a magnification of 20×. (c) Merged image. (d) Sectional TPM images obtained at depths of 90−150 μm at a magnification of 100×. Blue and yellow dots represent the nuclei and mitochondria, respectively.

**Figure S13.** (a, d, g) Sectional TPM image of the (a) normal, (d) adenoma (with low-grade dysplasia), and (g) adenocarcinoma co-labelled with **ABI-Nu** and **Pyr-CT** collected at Ch1 at a depth of 120 μm. (b, e, h) Sorted images in decreasing order of area. Count analysis was performed using Image-Pro® Analyzer 3D 7.0 software (Media Cybernetics). White marks in a, d, and g indicate the fluorescent regions identified by the software. (b, e, h) Dotted whited boxes include the fluorescent regions whose TPEF intensities were 90% of the maximum. (c, f, i) Bar diagram showing the distribution of the nuclei in decreasing order of area in b, e, and h. The vertical lines indicate regions where the TPEF intensity was 90% of the maximum.

**Figure S14.** (a, d, g) Sectional TPM image of the (a) normal, (d) adenoma (with low grade dysplasia), and (g) adenocarcinoma co-labelled with **ABI-Nu** and **Pyr-CT** collected at Ch2 at a depth of 120 μm. (b, e, h) Sorted images in decreasing order of area. Count analysis was performed using Image-Pro® Analyzer 3D 7.0 software (Media Cybernetics). White marks in a, d, and g indicate the fluorescent regions recognized by the software. (b, e, h) Dotted whited boxes include the fluorescent regions whose TPEF intensities were 90% of the maximum. (c, f, i) Bar diagram showing the distribution of the nuclei in decreasing order of area in b, e, and h. The vertical lines indicate regions where the TPEF intensity was 90% of the maximum.

**Figure S15.** (a, d, g) Sectional TPM image of the (a) normal, (d) adenoma (with low-grade dysplasia), and (g) adenocarcinoma co-labelled with **ABI-Nu** and **BF-MT** collected at Ch2 at a depth of 120 μm. (b, e, h) Sorted images in decreasing order of area. Count analysis was performed using Image-Pro® Analyzer 3D 7.0 software (Media Cybernetics). White marks in a, d, and g indicate the fluorescent regions identified by the software. (b, e, h) Dotted whited boxes include the fluorescent regions whose TPEF intensity was 90% of the maximum. (c, f, i) Bar diagram showing the distribution of the nuclei in decreasing order of area in b, e, and h. The vertical lines indicate the regions where the TPEF intensity was 90% of the maximum.

**Figure S16**. ^1^H NMR (300 MHz, acetone-*d*6) of compound **3**.

**Figure S17**. ^1^H NMR (300 MHz, DMSO) of compound **4**.

**Figure S18**. ^13^C NMR (100 MHz, DMSO) of compound **4**.

**Figure S19**. ^1^H NMR (300 MHz, CD_3_OD) of **ABI-Nu**.

**Figure S20**. ^13^C NMR (100 MHz, CD_3_OD) of **ABI-Nu**.

**Figure S21**. HRMS spectrum of **ABI-Nu**.

**Figure S22**. ^1^H NMR (500 MHz, CDCl_3_) of **Pyr-CT-AM**.

**Figure S23**. ^13^C NMR (100 MHz, CDCl_3_) of **Pyr-CT-AM**.

**Figure S24**. HRMS spectrum of **Pyr-CT-AM.**

**Figure S25**. ^1^H NMR (300 MHz, CDCl_3_) of compound **8**.

**Figure S26**. ^13^C NMR (100 MHz, CDCl_3_) of compound **8**.

**Figure S27**. ^1^H NMR (300 MHz, CDCl_3_) of compound **9**.

**Figure S28**. ^13^C NMR (100 MHz, CDCl_3_) of compound **9**.

**Figure S29**. ^1^H NMR (300 MHz, CDCl_3_) of compound **10**.

**Figure S30**. ^13^C NMR (100 MHz, CDCl_3_) of compound **10**.

**Figure S31**. ^1^H NMR (300 MHz, CDCl_3_) of compound **11**.

**Figure S32**. ^13^C NMR (100 MHz, CDCl_3_) of compound **11**.

**Figure S33**. ^1^H NMR (300 MHz, DMSO-*d*6) of **BF-MT**.

**Figure S34**. ^13^C NMR (100 MHz, DMSO-*d*6) of **BF-MT**.

**Figure S35**. HRMS spectrum of **BF-MT.**

**References**

1. Han, X. *et al*. Catalytic asymmetric syntheses of tyrosine surrogates. *J. Org. Chem.* 73, 8502-8510 (2008).

2. Ikeda, M., Nakagawa, H., Suzuki, T. & Miyata, N. Novel bisbenzimide-nitroxides for nuclear redox imaging in living cells. *Bioorg. Med. Chem. Lett.* 22, 1949-1952 (2002).

3. Kim, H. M. *et al*. Two-photon fluorescent turn-on probe for lipid rafts in live cell and tissue. *J. Am. Chem. Soc*. 13, 4246-4247 (2008).

4. Lim, C. S., Hong, S. T. Ryu, S. S., Kang, D. E. & Cho, B. R. Two-photon probes for lysosomes and mitochondria: simultaneous detection of lysosomes and mitochondria in live tissues by dual-color two-photon microscopy imaging. *Chem Asian J.* Epub ahead of print.

5. Drewry, J. A., Fletcher, S., Hassana, H. & Gunning, P. T. Novel asymmetrically functionalized bis-dipicolylamine metal complexes: peripheral decoration of a potent anion recognition scaffold. *Org. Biomol. Chem.* 7, 5074-5077 (2009).

6. (a) Long, J. R. & Drago, R. S. The rigorous evaluation of spectrophotometric data to obtain an equilibrium constant. *J. Chem. Ed.* 59, 1037 (1982). (b) Hirose, K. A practical guide for the determination of binding constants. *J. Incl. Phenom. Macrocycl. Chem.* 39, 193-209 (2001).
